# The effect of surface inclination and limb on knee loading measures in transtibial prosthesis users

**DOI:** 10.1186/s12984-019-0509-9

**Published:** 2019-03-12

**Authors:** Sean S. Doyle, Edward D. Lemaire, Julie Nantel, Emily H. Sinitski

**Affiliations:** 1 University of Ottawa, School of Human Kinetics, Montpetit Hall, 125 University, room 232, Ottawa, ON K1N 6N5 Canada; 2 University of Ottawa, Faculty of Medicine, Roger Guindon Hall, 451 Smyth Road, Ottawa, Ontario K1H 8M5 Canada; 3 Ottawa Hospital Research Institute, 505 Smyth Road, Ottawa, ON K1H8M2 Canada

**Keywords:** Knee, Biomechanics, Forces, Moments, Osteoarthritis, Slope, Cross-slope, Level

## Abstract

**Background:**

Osteoarthritis (OA) is a degenerative disease caused by the wearing of joint cartilage and bone. Literature has established that a prosthesis user’s intact limb is at greater risk of developing OA. This study analyzed the effect of commonly encountered surface inclinations on knee joint loading measures in able-bodied and transtibial prosthesis users.

**Methods:**

12 transtibial prosthesis users and 12 able-bodied participants walked across level ground, up slope, down slope, and cross slope (further divided into top and bottom slope depending on the location of the limb being analyzed). First and second peak external knee adduction moment (KAM), external knee adduction moment rate, and external knee adduction moment impulse were extracted from the stance phase of gait. Mixed ANOVA statistics with Bonferonni post hoc analyses were performed.

**Results:**

Significant limb differences were only found for KAM rate and first peak KAM. When compared to all other surfaces up slope had the significantly lowest KAM rate and was not significantly lower for all other tested variables. Down slope had significantly greater KAM rate than all surfaces except bottom slope. KAM second peak and KAM impulse analysis resulted in no significant differences.

**Conclusions:**

Individuals at risk for developing, or currently dealing with, knee OA could avoid walking for extended periods on down slope. Walking up moderate slopes may be considered as a complementary activity to level walking for rehabilitation and delaying OA progression.

The lack of significant limb differences suggests that second peak KAM and KAM impulse may not be appropriate load-related indicators of OA initiation among prosthesis users without OA. KAM rate was the most sensitive joint loading variable and therefore should be investigated further as an appropriate variable for identifying OA risk in individuals with transtibial amputations.

## Introduction

Osteoarthritis (OA) is a degenerative disease caused by the wearing of joint cartilage and bone. OA is the most frequent cause of disability in the United States, with the knee medial compartment being the most affected area [1]. Biomechanical risk factors for OA include obesity, trauma, muscular weakness, and overloading from varying sports and occupations [2]. Osteoarthritis is particularly relevant for people with lower limb amputations, where asymmetry and gait compensations can increase joint loads on the intact limb.

Altered walking mechanics and shifts in kinematic patterns caused by severe trauma, increased joint laxity, neuromuscular changes, and obesity can shift joint loading patterns to cartilage regions not suited for weight-bearing [3–5]. Lower limb prosthesis users have experienced severe trauma and consequently changed walking patterns. Unilateral transtibial prosthesis users may also have significant muscular strength discrepancies between limbs, with decreased force generation on the prosthetic side that contributes to a reliance on the intact limb [6–8]. Therefore, a lower limb prosthesis user’s intact limb is at greater risk of developing OA when compared to both the prosthetic limb [9–12] and able-bodied individuals [11, 13, 14].

Biomechanical OA research has focused on level walking joint loading patterns. External knee adduction moment (KAM) is an important knee loading measure, with larger KAM predicting OA onset and disease progression [15–19]. Although two adduction peaks occur during stance, the literature has focused mainly on the first peak in early stance. This peak is considered more important due to its larger magnitude [17, 20, 21] and steeper rise rate [12]. Whereas the first KAM peak for individuals with OA is typically greater than able-bodied controls, regardless of disease severity, the second peak is also greater than controls for individuals with more severe OA [17].

Recently, literature has begun to outline the importance of not only the KAM magnitude but also the rate with which this moment is applied. Faster loading rates cause both articular cartilage and subchondrial bone to become less deformable [22] and therefore more prone to damage. The importance of rate has been confirmed in animals where, while maintaining normal physiological loads, rapidly applied repetitive loading caused joint damage both in vitro [23] and in vivo [24].

Two variables that account for rate, maximum instantaneous slope from foot contact to the first peak of the KAM curve (KAM rate) and KAM impulse, the net positive impulse of the KAM curve, can identify individuals with OA. The KAM rate was associated with medial tibiofemoral joint degenerative changes using MRI, even after adjusting for KAM peak [25]. KAM impulse has been positively correlated with medial cartilage volume loss [26] and was more sensitive for discriminating between OA severity than KAM peak measurements [27]. Thorp et al. [28] reported differences, independent of confounding factors, between grade 2 and grade 3 OA severity patients that did not occur when only using KAM peak.

Whereas a reasonable understanding of knee OA biomechanical indicators exists for level walking, a better understanding of transtibial prosthesis user and able-bodied knee loading biomechanics across multiple terrains is needed to help advance clinical practice and prosthetic technology. Past research has shown that walking up an incline can decrease KAM [29, 30]; however, no research has been performed on the cross-slope and down slope surfaces for knee adduction joint loading variables. Significant asymmetries have been reported between limbs, even at a 6 degree cross slope [31]. The combination of different limb lengths and the need to keep the body vertical could alter knee adduction loading variables.

Therefore, this study investigated transtibial prosthesis user and able-bodied gait on level and sloped surfaces to determine how key knee load measures differ (KAM peak, KAM rate, KAM impulse). It was hypothesized that the prosthesis user group’s intact limb would have increased values for each joint loading variable when compared to both the prosthetic and able-bodied limbs due to the greater loads typically experienced during walking. It was also hypothesized that the cross-slope condition would exhibit the highest KAM variables.

## Methods

Twelve unilateral transtibial (TT) prosthesis users (79.9 ± 21.1 kg; 1.78 ± 0.07 m; 42 ± 10 years) and twelve height, sex, and weight matched able-bodied (AB) individuals (80.5 ± 11.8 kg; 1.78 ± 0.07 m; 39 ± 11 years) participated in the study (Table 1). In prosthesis users the intact limb is always the dominant limb and, in order to maintain the same data treatment for able-bodied individuals, AB dominant limb was defined as the preferred limb used to kick a ball. TT K-levels were three or four [32] and all prosthesis user participants wore their prosthesis daily and had no contralateral limb trauma or previous OA diagnosis. Participants were recruited through The Ottawa Hospital Rehabilitation Centre, Canadian Forces Health Services, Glenrose Rehabilitation Hospital, and the Ottawa community. All participants provided written informed consent and the study was approved by the appropriate ethics review boards. A previous power analysis using similar variables with transtibial prosthesis users determined a sample size of 8 was sufficient [12].

**Table 1:** Participant characteristics

Sex	Age (year)	Mass (kg)	Height (m)	Prosthetic Side	Etiology	Time since Amputation (years)	Level	Prosthetic Foot
M	63	63.0	1.83	L	Trauma	8	K4	Trias
M	36	96.4	1.80	R	Trauma	5	K4	C-walk
M	45	98.2	1.81	R	Trauma	8	K4	Re-Flex Rotate
M	35	67.3	1.73	R	Trauma	1	K4	Variflex
M	44	108.0	1.86	L	Trauma	1	K3	Variflex
M	50	109.0	1.85	R	Trauma	1	K4	Triton
M	56	87.3	1.80	L	Trauma	22	K3	Ceterus
M	45	47.3	1.73	R	Trauma	15	K4	Flex Foot
M	43	75.9	1.75	R	Osteosarcoma	0.7	K4	Re-Flex Rotate
M	37	81.8	1.80	R	Osteosarcoma	3	K4	Variflex
M	29	76.4	1.78	L	Congenital	28	K4	Variflex
F	27	47.7	1.60	R	Other	1	K3	Variflex

A simulated locomotor environment was provided by a CAREN (Computer Assisted Rehabilitation Environment)-Extended virtual reality system (MotekForce Link, Amsterdam, NL). CAREN consists of a motion capture system, embedded force plates, integrated treadmill, six degree of freedom motion platform, and a virtual scene. The CAREN system has been shown to produce results with negligible signal noise from treadmill operation when the platform is stationary [33].

Full body kinematics were tracked using a 6 degree-of-freedom, 57 marker set [34], sampled at 100 Hz, and platform motion was tracked with three markers. Ground reaction force data were sampled at 1000 Hz. Marker data was filtered using a 4th order low pass Butterworth filter with a 10 Hz cut-off frequency. Ground reaction force data used the same filter as the marker data with a 20 Hz cut-off frequency.

Participants had a 10–15 min CAREN acclimation period that included self-paced treadmill training and each encountered inclination. Self-paced mode was used for all trials on all inclinations [35]. The literature has shown self-paced mode to be an alternative to fixed-pace speeds that produces similar results [36]. Three trials were completed on a path through the virtual park scene, consisting of 20 m sections for eight separate walking conditions, for a total walking distance of 340 m per trial. All trials started from standing, followed by an initial level walking section, and concluded with a level walking section. All intermediate sections were randomized and separated by level sections. The conditions investigated in this paper were level (LG), down slope (DS: − 7° decline), up slope (US: + 7° incline, right cross slope (platform tilted 5°to the right), left cross slope (platform tilted 5°to the left). Cross slope trials were analyzed as top slope (TS) and bottom slope (BS), where TS occurred when the limb was at the top of the surface and BS when the limb was at the bottom (i.e., prosthetic limb TS would have the prosthetic limb at the top of the cross slope).

Marker and ground reaction force data were collected with the Vicon Nexus software and exported for processing within Visual3D. All data were extracted when the platform was stationary. First peak knee adduction moments (KAM-F) were extracted from the initial loading phase of stance (0–30% of gait cycle) and the second peak (KAM-S) was taken from the late phase of stance (40–80% of gait cycle). Peak knee extensor moment during early stance (0–30% of gait cycle) was also examined (KAM-E). Peaks were normalized to body weight to enable statistical comparisons between limbs (TT Prosthetic, TT Intact, AB Dominant, AB Non-dominant) and surfaces. KAM rate (KAM-R) was the maximum instantaneous slope of the normalized KAM first peak. KAM-I was the net positive impulse (time integral) of the KAM curve. Mixed ANOVA statistics with Bonferroni post hoc analyses were used for all statistical comparisons (*p* < 0.05). Treadmill speed (m/s) was added as a co-variate in the KAM-R and KAM-I statistical analyses to adjust for speed effects on study parameters.

## Results

KAM-F ANOVA analysis demonstrated significant main effects for limb (*F*(3, 217) = 3.817, *p* < 0.05, partial ƞ2 = 0.050) and surface (*F*(4, 217) = 5.870, *p* < 0.05, partial ƞ2 = 0.098), but no significant difference for the limb x surface interaction (Table 2). Intact limb KAM-F was significantly greater than the prosthetic limb, but not AB limbs. Up slope KAM-F was significantly less than level, down slope, and bottom slope.

**Table 2:** First peak knee adduction moment (Nm/kg) across 4 limbs and 5 surfaces. Standard deviation is in brackets

Limb	Level	Down Slope	Up Slope	Top Slope	Bottom Slope	Limb Overall
Dominant	0.57(0.14)	0.58(0.21)	0.39(0.11)	0.49(0.16)	0.56(0.13)	0.52(0.17)
Non-Dominant	0.52(0.15)	0.63(0.30)	0.40(0.14)	0.52(0.19)	0.59(0.25)	0.53(0.22)
Intact	0.56(0.19)	0.65(0.26)	0.43(0.23)	0.55(0.23)	0.64(0.20)	0.56(0.23)^‡^
Prosthetic	0.43(0.18)	0.44(0.24)	0.36(0.26)	0.48(0.19)	0.49(0.24)	0.44(0.22)
Surface Overall	0.52(0.17)*	0.57(0.26)*	0.40(0.19)	0.51(0.19)	0.57(0.21)*	

*significantly greater than up slope

‡ significantly greater than prosthetic limb

KAM-S ANOVA results showed no significant differences (Table 3). For KAM-R, significant main effects were found for both surface (*F*(4, 216) = 19.162, *p* < 0.05, partial ƞ2 = 0.262) and limb (*F*(3, 216) = 44.431, *p* < 0.05, partial ƞ2 = 0.382). Down slope had a significantly greater KAM-R when compared to all other surfaces except bottom slope (Table 4). Up slope was significantly less than all other surfaces. KAM-R on the prosthetic limb was significantly less than intact and AB limbs. The intact limb was significantly greater than prosthetic and AB limbs.

**Table 3:** Second peak knee adduction moment (Nm/kg) across 4 limbs and 5 surfaces. Standard deviation is in brackets

Limb	Level	Down Slope	Up Slope	Top Slope	Bottom Slope	Limb Overall
Dominant	0.38(0.17)	0.31(0.15)	0.24(0.17)	0.29(0.21)	0.32(0.19)	0.31(0.18)
Non-Dominant	0.36(0.09)	0.33(0.19)	0.33(0.18)	0.37(0.23)	0.43(0.25)	0.36(0.19)
Intact	0.36(0.12)	0.37(0.15)	0.30(0.14)	0.34(0.12)	0.39(0.18)	0.35(0.14)
Prosthetic	0.31(0.15)	0.28(0.11)	0.29(0.19)	0.30(0.16)	0.33(0.14)	0.30(0.15)
Surface Overall	0.35(0.13)	0.32(0.15)	0.29(0.17)	0.32(0.18)	0.37(0.19)

**Table 4:** First peak knee moment rate (Nm/kg/s) across 4 limbs and 5 surfaces. Standard deviation is in brackets

Limb	Level	Down Slope	Up Slope	Top Slope	Bottom Slope	Limb Overall
Dominant	15.27(3.55)	16.66(3.61)	9.44(2.07)	13.72(2.92)	16.59(3.92)	14.33(4.16)^†¥^
Non-Dominant	13.13(2.37)	15.77(4.63)	7.84(1.60)	13.19(2.35)	15.44(3.48)	13.07(4.13)^†¥^
Intact	15.68(3.90)	19.20(6.35)	11.06(2.63)	15.29(4.76)	17.08(4.66)	15.64(5.23)^¥^
Prosthetic	7.72(2.38)	9.77(4.55)	7.30(3.09)	8.62(3.17)	9.38(4.00)	8.56(3.54)^+^
Surface Overall	12.95(4.41)*^‡^	15.35(5.88)^‡^	8.91(2.77)*	12.74(4.09)*^‡^	14.57(4.99)^‡^	

*significantly less than down slope

‡ significantly greater than up slope

¥ significantly greater than the prosthetic limb

†significantly less than the intact limb

KAM-I analysis resulted in no significant differences for limb or surface (Table 5).

**Table 5:** First peak knee moment impulse (Nm/kg * s) across 4 limbs and 5 surfaces. Standard deviation is in brackets

Limb	Level	Down Slope	Up Slope	Top Slope	Bottom Slope	Limb Overall
Dominant	0.16(0.07)	0.14(0.07)	0.12(0.07)	0.14(0.08)	0.15(0.08)	0.14(0.07)
Non-Dominant	0.15(0.04)	0.16(0.09)	0.14(0.06)	0.15(0.08)	0.18(0.10)	0.16(0.08)
Intact	0.17(0.07)	0.16(0.08)	0.14(0.08)	0.15(0.06)	0.18(0.08)	0.16(0.07)
Prosthetic	0.15(0.08)	0.13(0.06)	0.13(0.10)	0.16(0.08)	0.15(0.07)	0.14(0.08)
Surface Overall	0.16(0.07)	0.15(0.08)	0.13(0.08)	0.15(0.07)	0.16(0.08)	

For peak knee extension moment (KAM-E), significant surface (*p* < 0.001, F(4,217) = 47.7, ƞ2 = 0.47) and limb (*p* < 0.001, F(3,217) = 124.1, ƞ2 = 0.63) main effects were observed (Table 6). A significant surface x limb interaction was also observed (*p* < 0.01, F(12,217) = 2.34, ƞ2 = 0.12). Further analysis revealed that the prosthetic limb had smaller KAM-E than intact and both AB limbs (*p* < 0.001), for all surfaces. Walking down slope increased KAM-E compared to all other surfaces for AB and intact limbs (*p* < 0.001). On the prosthetic limb, walking down slope increased KAM-E compared to level and up slope (*p* < 0.05). KAM-E was smaller on the non-dominant limb compared to dominant limb (*p* < 0.034), but was not significant when examining limb comparisons for each surface. KAM-E on the intact limb was not significantly different from AB limbs.

**Table 6:** Peak knee extension moment (Nm/kg) across 4 limbs and 5 surfaces. Standard deviation is in brackets. Significant surface x limb interaction was observed

Limb	Level	Down Slope	Up Slope	Top Slope	Bottom Slope	Limb Overall
Dominant	0.80 (0.17)	1.58 (0.29)*	0.94 (0.21)	0.82 (0.24)	0.93 (0.25)	1.01 (0.37)
Non-Dominant	0.69 (0.13)	1.39 (0.35)*	0.85 (0.16)	0.80 (0.27)	0.68 (0.27)	0.88 (0.35) ‡
Intact	0.68 (0.25)	1.44 (0.47)*	0.96 (0.29)	0.87 (0.24)	0.76 (0.23)	0.95 (0.40)
Prosthetic	0.16 (0.19) †	0.46 (0.29)** †	0.00 (0.23) †	0.16 (0.19) †	0.24 (0.25) †	0.21 (0.27) †
Surface Overall	0.59 (0.31)	1.22 (0.56)*	0.69 (0.46)	0.67 (0.37)	0.65 (0.35)	

*significantly greater than all other walking surfaces

*significantly greater than Level and Up Slope

† significantly less than intact and able-bodied limbs

‡ significantly less than dominant limb

## Discussion

This study analyzed five commonly encountered walking surfaces to determine if differences in OA related biomechanical variables existed for TT and AB groups. KAM variables related to knee OA were greater on various surfaces for AB and TT; however, significant limb differences were only found for KAM-R and KAM-F. These findings have implications on prosthetic design, rehabilitation strategies, quality of life, and OA physical activity recommendations.

When comparing AB non-dominant and dominant limbs, none of the frontal plane parameters resulted in significant differences. This finding was anticipated and further emphasizes the frontal plane differences observed in the TT group. The intact limb was expected to have significantly greater values for each of the knee loading variables since the OA incidence rate for the intact limb is much greater than both the prosthetic limb [9–12] and able-bodied limbs [11, 13, 14]. KAM-R was the only variable where intact limb results were significantly greater than prosthetic and both AB limbs, as hypothesized. These results suggest that KAM-R may be more sensitive, and thus a preferred variable when assessing knee load-related risk factors across multiple surfaces.

Currently, physical activity recommendations for individuals with OA advocate moderate, low-impact activity [37]. However these recommendations did not consider different loading consequences on different surfaces. Although not significant, walking uphill resulted in smaller values than the other surfaces, KAM-R was significantly less than all other surfaces, and KAM-F was significantly less than level, down and bottom slopes. Since excessive mechanical loading, or unloading, is essential to the onset and progression of OA [38, 39], walking up an incline may be a beneficial exercise for rehabilitation by reducing medial compartment loading. However, uphill walking requires greater knee extensor moments during stance compared to level walking [40, McIntosh, 2006]. Additionally, Lay et al. [40] reported increased extensor muscle activations at the knee when walking uphill. Quadriceps femoris weakness is considered one of the earliest symptoms of osteoarthritis [41, 42], and walking uphill could provide additional benefit of strengthening the knee extensors. Although peak knee extensor moment for uphill walking in this study was not significantly greater than the other walking surfaces (partially attributed to slower uphill walking speed), other factors such as ability and pain must be considered when selecting walking activities for rehabilitation.

The adduction moment was expected to be significantly greater on the cross-slope condition, with the bottom slope limb experiencing the highest values. However, no significant trends were found when comparing cross-slope to other surfaces, in agreement with Dixon and Pearsall [31] who reported that individuals increase joint moments at the ankle and hip, but not the knee, to compensate for the surface change in the coronal plane.

KAM-S and KAM-I analysis resulted in no significant differences for limbs or surfaces, suggesting that these outcome measures are not sensitive enough for assessing load-related OA risk factors. KAM-I values were similar to those previously established in the literature for lower limb amputees [43].

### First peak KAM

During level walking, KAM-F on the intact limb (0.56 Nm/kg) was similar to previously established values in the literature [12, 44, 45]. KAM-F on the intact limb was significantly greater that the prosthetic limb, but not the AB limbs. Therefore, our hypothesis that the intact limb would be significantly greater than prosthetic and AB limbs was only partially supported. Previous research reported increased KAM-F during the intact limb’s initial loading phase [44] and that the intact limb is more likely to develop knee OA when compared to controls [46]. However, a literature review by Foroughi, Smith, & Vanwanseele [47] suggested that KAM may only be an indicator of disease progression and not initiation, since only two of the included studies found a significant difference between controls and individuals with low severity OA. They concluded that KAM-F was unlikely to be a risk factor for OA development. The transtibial prosthesis user participants in this study had no history of OA and, if KAM-F is only an indicator of disease progression, no significant difference between the limbs should have been found. Therefore, the findings from this study contradict the literature review and would suggest that KAM-F should be investigated as a risk factor for OA development in lower limb prosthesis users.

### Second peak KAM

Past literature outlined the importance of KAM-F due to its larger magnitude [17, 20, 21] and steeper rise rate [12]. KAM-S could help identify disease severity since individuals with more severe OA have shown differences when compared to AB individuals [17]. However, since KAM-S showed no significant differences across all tested variables, further research is needed to verify KAM-S as a predictive factor for OA onset in lower limb prosthesis users. The KAM-S variables extracted from this study were lower than previously established values in the literature with similar non-significant findings when comparing different prosthetic foot types [48].

### KAM rate

KAM-R was the only variable where intact limb results were significantly greater than the prosthetic and AB limbs, and prosthetic limb results were significantly less than intact and able-bodied limbs. This finding supported our hypothesis that results would be greater on the intact limb than the prosthetic and AB limbs [9–12]. Morgenroth et al. [25] found that KAM-R was greater for participants with increased medial tibiofemoral joint degenerative changes, even after adjusting for KAM peak, and reported no significant increase in KAM-I. The results of this study agreed with Morgenroth et al. and KAM-R may be the most sensitive measure of the four examined. A study by Lloyd et al. [12] produced notably lower KAM-R when compared to this study. However the participants in this study also exhibited higher peak KAM and KAM-R was normalized to mass. These differences could account for the KAM-R differences between studies.

Down slope walking had significantly greater KAM-R than all other surfaces except bottom slope. This suggested that people at risk of developing OA may want to avoid activities such as hiking with excessive slope changes. Further studies should aim to identify factors that contribute to eccentric control of the prosthetic limb, or enhance prosthetic design to decrease the reliance on the intact limb. However, uphill KAM-R was significantly less than all other surfaces. Uphill slope walking may be beneficial for rehabilitation to ease the development of OA while still maintaining an acceptable level of physical activity. This recommendation is in agreement with the literature that advocates walking on gradients greater than ten [30] and 12 % [49]. Walking uphill may be beneficial for individuals who can tolerate the greater knee extension moment requirements (i.e., if the person maintains the same uphill walking speed).

### Limitations

Other measures not included in this research have been proposed as alternatives to KAM-F. Adouni and Shirazi-Adi [50] suggested that knee adduction angle is a better indicator of tibiofemoral compartment loading when compared to adduction moment. A drop of 1.5 degrees in adduction angle was found to decrease medial contact force by 12% as opposed to a 4% decrease when the adduction moment is reduced by half. Chehab et al. [51] concluded that external peak knee flexion moment should also be considered when assessing the risk for OA progression. However, the literature has also shown that there is no relationship between peak knee flexion moment and OA progression measures [52]**.** Further investigation should be performed on other potential biomechanical indicators of both OA initiation and progression to determine the surface effects on these indicators.

The incidence rates of medial compartment OA in prosthesis users is currently not well established in the literature. It is possible that, unlike able-bodied individuals, the medial compartment is not the most affected area. However, a study by Melzer et al. [13] determined that the knee medial compartment was the most affected site on the contralateral limb. It is also possible that OA initiation in amputees is not joint load related and could be chemically-mediated or related to other factors such as neuromuscular changes.

The study findings may not be generalizable to all functional levels of lower limb prosthesis users. Individuals with a lower functional level may rely more on the intact limb, making KAM discrepancies more severe.

Finally, due to the selection of a Bonferroni post-hoc test the results are conservative and should be interpreted as such. A Bonferroni was chosen in order to control for per-family error rate as well as familywise error rate.

## Conclusion

Walking on certain surfaces encountered in daily life results in greater values for variables related to OA. Therefore, individuals at risk for developing, or currently dealing with, knee OA should avoid walking for extended periods on down slopes. Up slope had the significantly lowest KAM-R and trended lower for all other tested variables when compared to all other surfaces. This suggested that walking up slope may be considered as a complementary activity to level walking for rehabilitation and delaying OA progression for individuals who are able to tolerate the greater knee extension moments required.

Individuals with amputations are at a greater risk of developing OA in the contralateral limb [9–14] and it was hypothesized that the intact limb would have greater values for each of the OA indicator variables when compared to the prosthetic and AB limbs. The lack of significant limb differences for KAM-S and KAM-I suggests these parameters may not be appropriate load-related indicator of OA initiation among prosthesis users without OA. KAM-R was the most sensitive variable and therefore may be the most appropriate variable for identifying OA risk in individuals with transtibial amputations.

## Abbreviations

AB: Able-Bodied
BS: Bottom slope
CAREN: Computer Assisted Rehabilitation Environment
DS: Down slope
KAM: Knee adduction moment
KAM-F: First peak knee adduction moment
KAM-I: Knee adduction moment impulse
KAM-R: Knee adduction moment rate
KAM-S: Second peak knee adduction moment
LG: Level ground
OA: Osteoarthritis
TS: Top slope
TT: Transtibial
US: Up slope
